# Omega-3 Polyunsaturated Fatty Acid Eicosapentaenoic Acid or Docosahexaenoic Acid Improved Ageing-Associated Cognitive Decline by Regulating Glial Polarization

**DOI:** 10.3390/md21070398

**Published:** 2023-07-10

**Authors:** Juan Xia, Longen Yang, Chengyi Huang, Shuyi Deng, Zhiyou Yang, Yongping Zhang, Cai Zhang, Cai Song

**Affiliations:** 1 Research Institute for Marine Drugs and Nutrition, College of Food Science and Technology, Guangdong Ocean University, Zhanjiang 524088, China; 2 Guangdong Provincial Key Laboratory of Aquatic Product Processing and Safety, College of Food Science and Technology, Guangdong Ocean University, Zhanjiang 524088, China; 3 Marine Medicine Research and Development Center, Shenzhen Institutes of Guangdong Ocean University, Shenzhen 518120, China

**Keywords:** ageing, memory impairment, glial polarization, n-3 PUFAs

## Abstract

Neuroinflammation induced by microglial and astrocyte polarizations may contribute to neurodegeneration and cognitive impairment. Omega (n)-3 polyunsaturated fatty acids (PUFAs) have anti-inflammatory and neuroprotective effects, but conflicting results were reported after different n-3 PUFA treatments. This study examined both the change in glial polarizations in ageing rats and the differential effects of two omega-3 PUFAs. The results showed that both PUFAs improved spatial memory in ageing rats, with docosahexaenoic acid (DHA) being more effective than eicosapentaenoic acid (EPA). The imbalance between microglial M1/M2 polarizations, such as up-regulating ionized calcium binding adaptor molecule 1 (IBA1) and down-regulating CD206 and arginase-1 (ARG-1) was reversed in the hippocampus by both n-3 PUFAs, while the DHA effect on CD206 was stronger. Astrocyte A1 polarization presented increasing S100B and C3 but decreasing A2 parameter S100A10 in the ageing brain, which were restored by both PUFAs, while DHA was more effective on S100A10 than EPA. Consistent with microglial M1 activation, the concentration of pro-inflammatory cytokines tumor necrosis factor (TNF)-α, interleukin (IL)-1β and IL-6 were significantly increased, which were attenuated by DHA, while EPA only suppressed IL-6. In correlation with astrocyte changes, brain-derived neurotrophic factor precursor was increased in ageing rats, which was more powerfully down-regulated by DHA than EPA. In summary, enhanced microglial M1 and astrocytic A1 polarizations may contribute to increased brain pro-inflammatory cytokines, while DHA was more powerful than EPA to alleviate ageing-associated neuroimmunological changes, thereby better-improving memory impairment.

## 1. Introduction

Ageing is a causative factor for the development of neurodegenerative diseases like cognitive decline and Alzheimer’s disease (AD) [1]. Neuroanatomical studies reported that hippocampal atrophy contributes to learning and memory impairment, and the inducers could be neuroinflammation and a reduction in neurogenesis and synaptic plasticity [2]. Microglia and astroglia, the vital central immune cells can be activated into inflammatory or neurotoxic phenotypes (M1 microglia and A1 astrocytes) or neuroprotective types (M2 microglia and A2 astrocytes) [3]. Ageing-induced chronic low-grade inflammation promotes microglia into a sensitized, reactive, or primed pro-inflammatory M1 phenotype [4,5], which increased the release of pro-inflammatory factors, such as tumor necrosis factor (TNF)-α, interleukin (IL)-1β, and IL-6, and decreased the expression of anti-inflammatory M2 microglia marker arginase 1(ARG-1) and CD206, as well as anti-inflammatory cytokines IL-4 and IL-10 [6]. As a consequence of microglial M1 activation, these pro-inflammatory cytokines thereafter trigger astrocyte A1 polarization. The increased A1 marker C3 and decreased A2 neurotrophic factor brain-derived neurotrophic factor (BDNF) led to neurotransmitter deficiency and neuronal apoptosis [7]. However, the neuroinflammatory mechanisms mediated by glial polarization imbalance associated with ageing-induced memory impairment were not fully understood.

As mentioned above, glial polarizations may contribute to neurodegeneration and cognitive impairment. The regulation of glial function may provide a novel therapeutic direction to alleviate ageing-related neurodegenerative disease. Omega (n)-3 polyunsaturated fatty acids (PUFAs), indispensable components of cell membranes, play an important role in maintaining the membrane structural integrity and fluidity of immune and neuronal cells. The synthesization of n-3 PUFAs is restricted in human bodies but is mainly obtained from marine sources [8]. n-3 PUFAs reduction has been reported in the physiological processes of ageing, which is associated with brain atrophy and memory impairment [9,10]. We and others have reported that n-3 PUFAs eicosapentaenoic acid (EPA) or docosahexaenoic acid (DHA) supplementation exerted preventative, ameliotropic, and/or neuroprotective effects on psychiatric and neurological disorders, such as depression [11] and Alzheimer’s disease [12,13]. The mechanism by which n-3 PUFAs effectively improved these diseases was through inhibiting the expression of microglia M1 phenotype marker CD11b and inducible nitric oxide synthase (iNOS) and downregulating the level of proinflammatory cytokines IL-1β, TNF-α and IL-6, while up-regulating the microglia M2 phenotype marker CD206 and anti-inflammatory cytokines IL-10 [14]. After n-3 PUFAs balance microglia M1 and M2 polarizations, the damage from neuroinflammation on neurons was alleviated, which was mainly involved in nuclear factor kappa-B (NF-κB) pathway inhibition [15]. Similarly, n-3 PUFAs suppress the pro-inflammatory and toxic astroglia A1 phenotype polarizations by decreasing the expression of C3 and S100B [16] but promote astrocyte differentiation from neural stem cells accompanied by an increase in neurotrophic factors BDNF and glial cell line-derived neurotrophic factor (GDNF) [17]. However, DHA was reported to be better in terms of its neuroprotective effect by promoting neuronal survival, synaptic vesicle fusion, neurotransmitter releases and neuronal plasticity [18]. Differently, we and others reported that EPA but not DHA markedly attenuated mental-disorder-associated and inflammation-induced cognitive impairment. However, whether and how EPA and DHA treatments modulate glial polarizations and improve memory impairment in the ageing brain is still unknown. Thus, the present study aimed to determine whether the imbalance between two types of glial polarizations is associated with ageing-induced memory impairment and compare and evaluate the therapeutic effects of n-3 PUFAs DHA and EPA on memory impairment and their underlying neuroimmune mechanisms.

## 2. Results

### 2.1. Memory and Locomotor Impairment Occurred in Ageing Rats, Which Was Better Improved by DHA Than EPA

**Figure 1:**
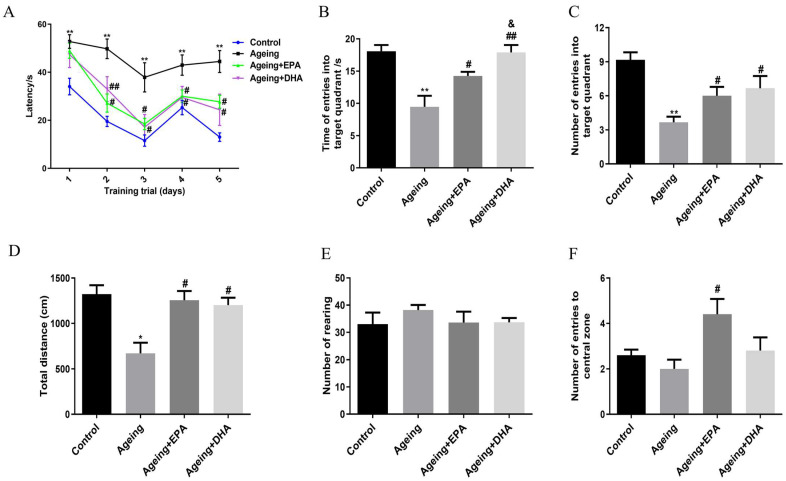
Memory and locomotor impairment that occurred in ageing rats was better improved by DHA than by EPA. The latency time (**A**), time (**B**) and number (**C**) of entries into the target quadrant in MWM behavioral test. The total distance (**D**), number of rearing (**E**) and entries into the central zone (**F**) in the OFT. The data were expressed as mean ± SEM (*n* = 12 in control group, *n* = 9 in other three groups). * *p* < 0.05, ** *p* < 0.01 versus control group; # *p* < 0.05, ## *p* < 0.01 versus ageing group. & *p* < 0.05 versus ageing + EPA group.

In the Morris water maze (MWM) test, the ageing rats showed increased latency to find the platform (Figure 1A) and decreased time spent on and numbers in the target quadrant (*p* < 0.01) (Figure 1B,C), indicating significant memory impairment in ageing rats when compared to the control group. Even though both EPA and DHA reversed memory impairment in ageing rats by decreasing the latency to the platform (*p* < 0.05) (Figure 1A) and increasing the time spent and the number of entries into the target quadrant (*p* < 0.05) in ageing rats (Figure 1B,C), rats fed DHA showed longer time spent in the target quadrant than the EPA (*p* < 0.05) (Figure 1B). In the open field test (OFT), the total locomotor distance was decreased in ageing rats compared with the control (*p* < 0.05), which was improved by EPA (*p* < 0.05) or DHA (*p* < 0.05) treatment (Figure 1D). EPA treatment was more powerful than DHA in the increase in central entries compared with ageing rats (Figure 1F). However, there were no differences between controls and ageing rats in the number of rearing and center entries (Figure 1E,F).

### 2.2. n-3 and n-6 PUFAs Imbalance Were Both Improved by DHA and EPA

**Figure 2:**
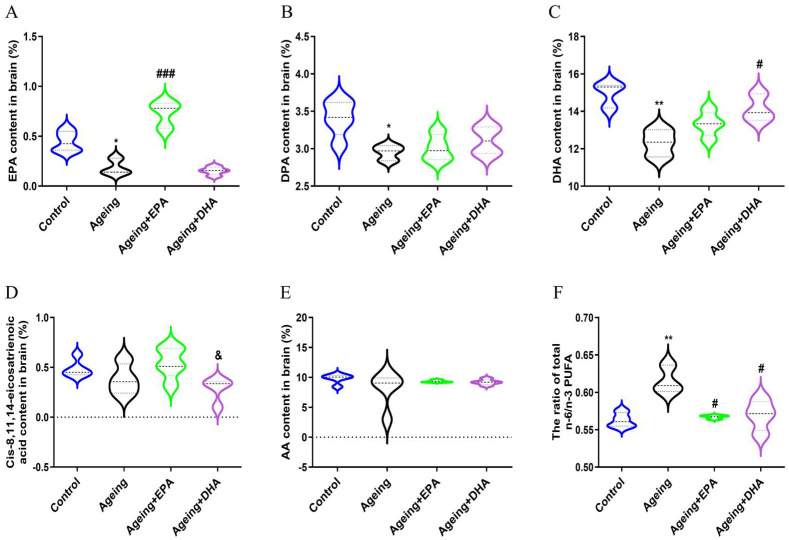
The increase in n-6/n-3 PUFAs ratio in ageing rats was effectively reversed by EPA and DHA. The contents of n-3 PUFAs EPA (**A**), DPA (**B**), and DHA (**C**) and contents of n-6 PUFAs cis-8,11,14-eicosatrienoic acid (**D**) and AA (**E**) as well as n-6/n-3 PUFAs ratio (**F**). The data were expressed as mean ± SEM (*n* = 5). * *p* < 0.05, ** *p* < 0.01 versus control group; # *p* < 0.05, ### *p* < 0.001 versus ageing group. & *p* < 0.05 versus ageing + EPA group.

Compared with the control group, significantly reduced contents of n-3 PUFAs EPA (*p* < 0.05), DPA (*p* < 0.05), and DHA (*p* < 0.01) (Figure 2A–C) were found in ageing rat brains, without changing n-6 PUFAs cis-8,11,14-eicosatrienoic acid and arachidonic acid (AA) concentrations (Figure 2D,E). The ratio of n-6/n-3 PUFAs was markedly increased in ageing rats compared with the control group (*p* < 0.01) (Figure 2F). Supplementation of EPA or DHA elevated the level of EPA (*p* < 0.001) or DHA (*p* < 0.05), respectively (Figure 2A,C), and both EPA and DHA treatments significantly reduced the ratio of n-6/n-3 PUFAs in ageing rats (*p* < 0.05) without an effect on DPA and n-6 PUFA contents (Figure 2B,D–F).

### 2.3. Abnormal Microglial M1 and M2 Polarizations in the Hippocampus of Ageing Rats Was Better Ameliorated by DHA Than EPA through Upregulating CD206

**Figure 3:**
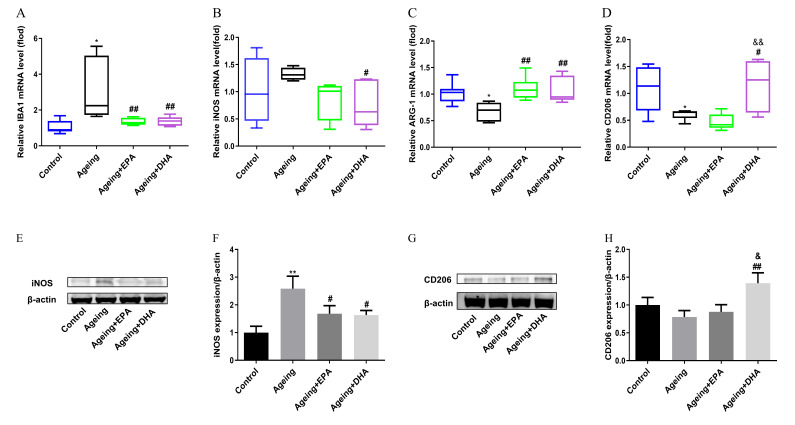
Imbalance between microglial M1 and M2 polarization that occurred in ageing rats was attenuated by EPA and DHA. The mRNA expression of IBA1 (**A**), iNOS (**B**), ARG-1 (**C**), and CD206 (**D**). The protein expression of iNOS (**E**,**F**) and CD206 (**G**,**H**). The data were expressed as mean ± SEM (*n* = 6). * *p* < 0.05, ** *p* < 0.01 versus control group; # *p* < 0.05, ## *p* < 0.01 versus ageing group. & *p* < 0.05, && *p* < 0.01 versus ageing+ EPA group.

Compared with the control group, mRNA expression of M1 microglia markers ionized calcium binding adaptor molecule 1 (IBA1) (*p* < 0.05) was upregulated, while the mRNA expression of M2 microglia marker CD206 (*p* < 0.05) and ARG-1 (*p* < 0.05) was downregulated in the hippocampus of ageing rats (Figure 3A,C,D). Either EPA or DHA treatment significantly inhibited M1 microglial and restored M2 microglial polarization (Figure 3A–D). Nevertheless, DHA was better in the upregulation of CD206 mRNA expression than EPA (Figure 3D). Increased protein expression of iNOS (*p* < 0.01) was found in the ageing hippocampus, which was attenuated by either EPA or DHA (*p* < 0.05) (Figure 3E,F). DHA rather than EPA treatment significantly upregulated CD206 protein expression compared with ageing rats (*p* < 0.01) (Figure 3G,H).

### 2.4. Neuroinflammation in Ageing Rats Were Both Inhibited by DHA and EPA

**Figure 4:**
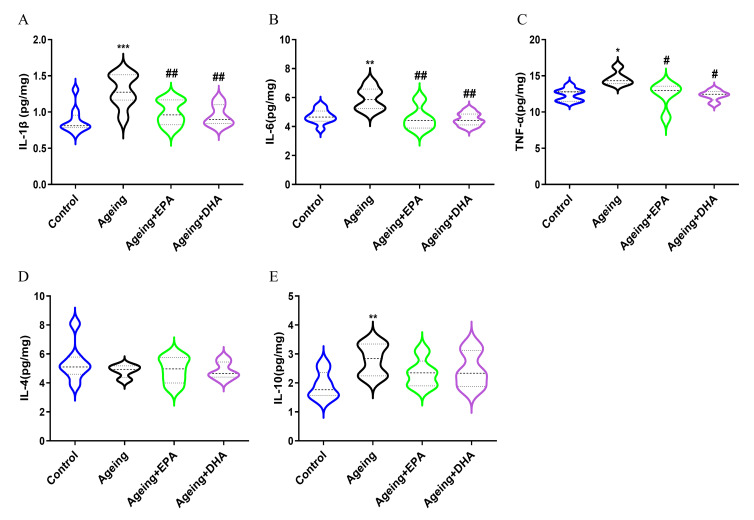
Neuroinflammatory responses upregulated during normal ageing were inhibited by EPA and DHA. The concentrations of pro-inflammatory cytokines IL-1β (**A**), IL-6 (**B**), and TNF-α (**C**). The concentrations of anti-inflammatory cytokines IL-4 (**D**) and IL-10 (**E**). * *p* < 0.05, ** *p* < 0.01, *** *p* < 0.001 versus control group; # *p* < 0.05, ## *p* < 0.01 versus ageing group.

Compared with the control group, the concentration of pro-inflammatory cytokines IL-1β, IL-6, and TNF-α were significantly increased in the hippocampus of ageing rats (IL-1β, *p* < 0.001; IL-6, *p* < 0.01; TNF-α, *p* < 0.05) (Figure 4A–C), which was equally reversed by EPA or DHA treatment (*p* < 0.05) (Figure 4A–C). Meanwhile, the anti-inflammatory cytokine IL-10 was significantly upregulated (*p* < 0.01), without changing the IL-4 concentrations (Figure 4D,E). Neither EPA nor DHA had a significant effect on these anti-inflammatory cytokine concentrations in ageing rats (Figure 4D,E).

### 2.5. Abnormal Astroglia A1/A2 Phenotypic Polarizations in the Hippocampus of Ageing Rats Were Both Attenuated by EPA and DHA

**Figure 5:**
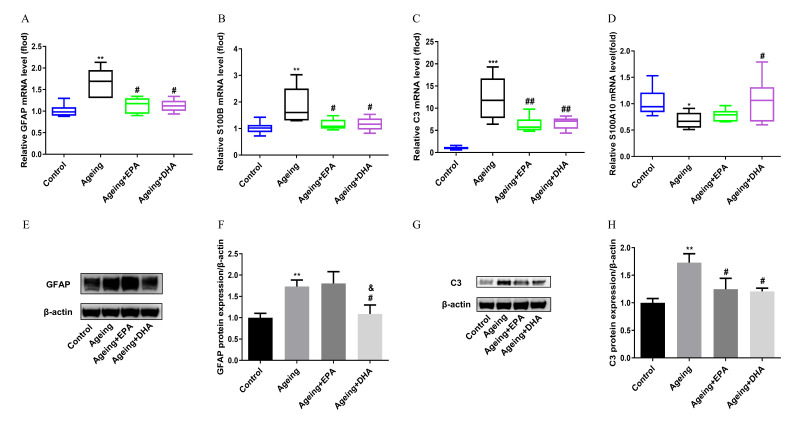
Astroglia polarization imbalance induced in the ageing rat hippocampus was reversed by EPA and DHA. The mRNA expressions of GFAP (**A**), S100B (**B**), C3 (**C**), and S100A10 (**D**). The WB bands of marker proteins (**E**,**G**), and the relative protein expression of GFAP (**F**) and C3 (**H**). The data were expressed as mean ± SEM (*n* = 6). * *p* < 0.05, ** *p* < 0.01, *** *p* < 0.001 versus control group; # *p* < 0.05, ## *p* < 0.01 versus ageing group; & *p* < 0.05 versus ageing + EPA group.

The mRNA expressions of activated astroglia marker glial fibrillary acidic protein (GFAP) (*p* < 0.01), A1 marker S100B (*p* < 0.01) and C3 (*p* < 0.001) were significantly upregulated, while A2 marker S100A10 (*p* < 0.05) was markedly downregulated (Figure 5A–D) in the ageing hippocampus. Both GFAP and C3 changes were confirmed at the level of protein expression. EPA and DHA equally inhibited the mRNA expression of GFAP (*p* < 0.05), S100B (*p* < 0.05) and C3 (*p* < 0.01)in the hippocampus of ageing rats (Figure 5A–C). Surprisingly, the decrease in mRNA expression of astroglia A2 marker S100A10 and the increase in protein expression of GFAP were significantly attenuated by DHA treatment, but not EPA (*p* < 0.05) (Figure 5D–F).

### 2.6. Activation of proBDNF-p75(NTR) in the Hippocampus of Ageing Rats Was Inhibited by EPA and DHA

**Figure 6:**
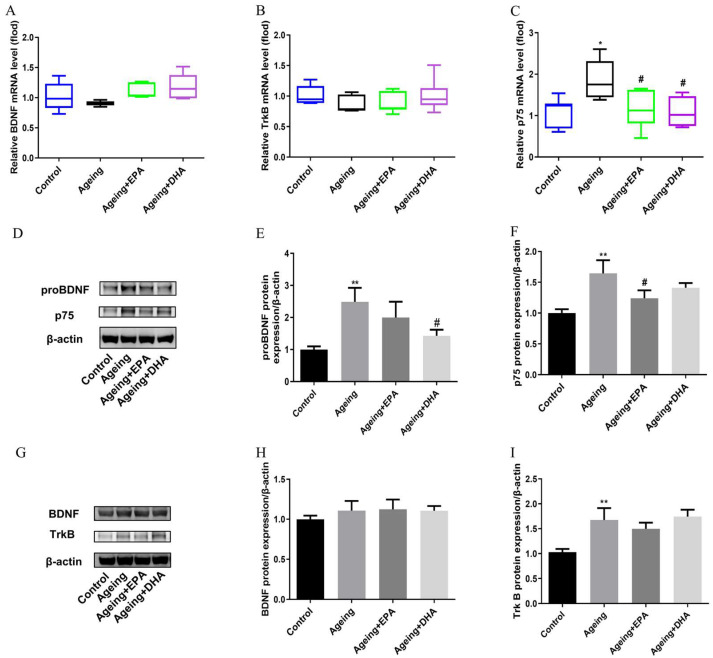
The dysregulation of neurotrophic factors and receptors that occurred in ageing rats was improved by EPA and DHA. The mRNA expression of BDNF (**A**), TrkB (**B**), and p75 (**C**). The WB bands of protein (**D**,**G**), and the relative protein expression of proBDNF (**E**), p75 (**F**), BDNF (**H**), and TrkB (**I**). The data were expressed as mean ± SEM (*n* = 6). * *p* < 0.05, ** *p* < 0.01 versus control group; # *p* < 0.05 versus ageing group.

Compared with the control group, mRNA expression of BDNF and its high-affinity receptor tropomyosin-related kinase B (TrkB) were not changed, while p75 receptor mRNA expression (*p* < 0.05) was significantly increased in ageing rats (Figure 6A–C). EPA and DHA had no significant effects on BDNF and TrkB mRNA expression; however, both DHA and EPA indiscriminately reduced the mRNA expression of p75 (*p* < 0.05) (Figure 6A–C). Importantly, the expression of BDNF precursor protein proBDNF (*p* < 0.01) and its receptor p75 (*p* < 0.01) were significantly upregulated in the hippocampus of ageing rats (Figure 6D–F). Both DHA or EPA inhibited the expression of proBDNF (*p* < 0.05) or p75 receptor (*p* < 0.05) (Figure 6D–F). In concert with mRNA expression, no change in BDNF protein expression occurred between control and ageing animals, while TrkB protein was upregulated in ageing rats compared with controls (*p* < 0.05) (Figure 6G–I). EPA and DHA did not affect BDNF/TrkB, but normalized proBDNF/p75 protein expressions in ageing rats.

## 3. Discussion

In this study, the possible mechanism by which the imbalance between different glial cell polarizations may contribute to ageing-associated cognitive decline was first explored. Growing evidence has suggested different actions of EPA and DHA in the treatment of psychiatric and neurodegenerative diseases. Then, the effects of different n-3 PUFAs on cognitive impairment and related glial polarizations in the ageing brain were studied. The current study distinguished different effects between EPA and DHA and revealed some possible mechanisms in the treatment of ageing-associated cognitive decline. Several new findings were discussed as follows:

First, the results showed that both spatial learning and memory in MWM and exploration in OFT were impaired in normal ageing rats compared to adult rats. Meanwhile, the concentration of brain n-3 PUFAs, such as EPA, DPA and DHA, were decreased in ageing brains. A longitudinal cohort study of elderly Japanese with cognitive decline over 10 years displayed decreased serum DHA, which was positively correlated with cognitive decline [19]. Thus, DHA supplementation was reported to improve cognitive decline in older adults with mild cognitive impairment [20,21] or animal models of AD [22]. Most studies were based on supplementing a mixture of EPA and DHA, whereas the present study compared the effects of EPA and DHA on memory impairment in ageing animals. This study demonstrated that both EPA and DHA could improve the ageing-related memory impairment in the MWM test, while DHA effects were better than EPA in terms of increasing the time of entries into the target quadrant. However, a previous study reported that both EPA and DHA indiscriminately exhibited anti-ageing effects by antioxidation and reducing ageing-related proteins [23]. Since EPA supplementation or its derivatives could significantly increase the amount of EPA and DHA in the brain [24,25], EPA’s effect may partly depend on DHA function. The PUFAs profile in our study showed that both EPA and DHA treatments respectively reversed the decrease of EPA or DHA in ageing rats’ brains, which was more conducive to revealing their commonalities and specific mechanisms for improving ageing-related memory impairment.

Secondly, ageing-associated chronic low-grade inflammation is one of the contributors to cognitive deficits, which was mainly triggered by activated microglia [26]. Numerous studies have reported the activation of the microglia M1 phenotype in normal ageing, which upregulated feature genes IBA1 and iNOS and produced pro-inflammatory cytokines IL-1β, IL-6, and TNF-α [27,28,29]. The alternative M2 phenotype exhibits anti-inflammatory activity with the expression of representative markers ARG-1 and CD206, along with secreted anti-inflammatory cytokines IL-4 and IL-10 [30]. Thus, shifting M1 polarization to M2 might be a potential therapy for neurodegenerative diseases. The anti-neuroinflammatory activities of EPA and DHA attributed to the balance between M1 and M2 polarization had been confirmed to improve depression [31], Alzheimer’s disease [32], Parkinson’s disease [33] and other neuropsychiatric disorders by our and others’ group, which are consistent with the findings in the current study. Both EPA and DHA reversed the increased M1 marker IBA1 mRNA and iNOS protein expression and decreased M2 marker ARG-1 mRNA expression in the hippocampus of ageing rats, while DHA showed stronger effects than EPA in terms of increasing the expression of CD206. An important finding that supports our results was that DHA promoted the expression of CD206, M2 phenotype in human CHME3 microglia cells, but not EPA [32]. The promotion of M2 macrophage polarization by DHA primarily relied on the modulation of the p38 MAPK signaling pathway and autophagy [34]. CD206 plays an important role in the procedure of anti-neuroinflammation as an M2 phenotype marker of microglia. Previous studies have demonstrated that the therapeutic effects of DHA on traumatic brain injury depend on increasing CD206-positive phagocytic microglia [35]. Thus, CD206 might play a critical neuroprotective role in DHA-related cognitive improvement, which deserves further research. Furthermore, both DHA and EPA equally inhibited the production of pro-inflammatory cytokines IL-1β, IL-6, and TNF-α in the hippocampus of ageing rats, without changing anti-inflammatory cytokines IL-4 and IL-10.

Similar to microglia, astroglia depolarize to two phenotypes, A1 and A2. A2 plays supporting and maintaining roles for brain homeostasis in normal conditions, which could be damaged by neuroinflammation [36]. Transcriptional analysis for astroglia in ageing mice showed that the increase in astroglia A1 pro-inflammatory or cytotoxic response phenotype could drive an ageing-related cognitive decline [7]. The present study further demonstrated ageing-dependent remodeling of astroglia, with increased GFAP and A1 marker genes S100B and C3 complement and decreased protective A2 phenotype marker gene S100A10. Both EPA and DHA inhibited C3 expression in the ageing brain, while DHA, interestingly, was more effective than EPA in reducing GFAP but upregulating A2 marker S100A10 transcription. GFAP, a common marker of astroglia, was generally elevated in rodents and humans during ageing [37], which indicates a potential marker of cognitive impairment induced by ageing [38], AD [39], heart failure [40] and type 2 diabetes [41]. Previous studies showed that DHA regulated glial cell polarization by activating PPARγ in AD [42] and enhanced the expression of GFAP by the up-regulation of both PI3K/AKT-dependent FABP7–PPARγ interaction and MKP3 in astrocytes in young rat brains [43]. Conversely, and excitingly, the present study showed, for the first time, the specific inhibition of DHA on GFAP in ageing rats, which was supported by a similar effect observed in a chronic constriction injury [44].

Thirdly, BDNF, a neurotrophin produced by neurons and astroglia, plays a crucial role in maintaining neurogenesis and neuronal plasticity, the alteration of which was related to cognitive deficits in ageing and AD [45]. There are two BDNF receptors; the first is the high-affinity TrkB receptor, which promotes cell survival, neuronal growth, synaptic plasticity and neurotransmitter release. Exogenous BDNF inhibits ageing-activated microglia through the TrkB-Erk-CREB pathway [46]. The other is p75 receptor, which promotes cell apoptosis and inhibits cell survival through binding to proBDNF. In contrast, some studies reported no relationship between ageing-associated neurodegeneration and mRNA expression of BDNF or TrkB receptors in the hippocampus [47]. Even though the present study confirmed that no significant changes were observed in mRNA and protein expression of BDNF between the ageing and control rats, TrkB was increased in ageing rats. Unexpectedly, EPA and DHA had no effects on BDNF and TrkB expression in ageing rats. However, as reported previously, proBDNF, the precursor protein of BDNF, with an opposite effect on neuronal plasticity through binding to the p75 receptor, was increased in the hippocampus, which mainly contributes to ageing-related memory impairments [48]. Consistently, the present study showed that protein expression of proBDNF and its receptor p75 were elevated in ageing rats, while both EPA and DHA decreased the mRNA expression of p75. More interesting, the present study, for the first time, found that EPA and DHA exhibited different and specific effects on the protein expression of these factors as DHA downregulated proBDNF protein, while EPA downregulated p75 protein.

In summary, the present study demonstrated that cognitive decline occurred during the progress of normal ageing, the mechanisms of which mainly involved an imbalance between microglial M1 and M2, and astroglia A1and A2 phenotypic polarizations, as well as the upregulation of proBDNF and p75 signaling. In addition, the decrease in endogenous n-3 PUFAs in ageing progression and improvement of n-3 PUFAs on memory deficit, undoubtedly, provided a new chance for the prevention or treatment of ageing-related cognitive disorders. Both EPA and DHA improved the ageing-related learning and memory impairment, while DHA effects were more pronounced than EPA. The common mechanisms of EPA or DHA were as follows: (1) both EPA and DHA restored the ratio of the n-3/n-6 PUFAs profile; (2) EPA and DHA equally inhibited the ageing-related neuroinflammation induced by M1 microglia excessive activation; (3) EPA and DHA both inhibited astroglia A1 phenotype by decreasing C3 and S100B expression; (4) both DHA and EPA effectively suppressed the proBDNF/p75 pathway without changing BDNF-TrkB signaling. Furthermore, differences between EPA and DHA were revealed: (1) DHA was superior to EPA in promoting M2 microglial polarization; (2) DHA was more able than EPA to block GFAP expression while promoting A2 polarization. Collectively, compared with EPA, DHA is preferable for treating ageing-related cognitive and memory impairments.

## 4. Materials and Methods

### 4.1. Animals and Experimental Procedure

Twelve adult male (3-month old) and twenty-seven male ageing (24-month old) Sprague–Dawley (SD) rats were housed as two per cage in a standard environment with 23 ± 1 °C and relative humidity 50 ± 10% under a regular 12 h light/dark cycle (light on 7:00 A.M.). The experimental process was performed according to the National Institutes of Health Guide for the Care and Use of Laboratory Animals and approved by the Animal Ethics Committee of Guangdong Ocean University.

Adult rats were used as a control while the ageing rats were divided into three groups: ageing (saline), ageing + EPA (500 mg/kg/day EPA) and ageing + DHA (500 mg/kg/day DHA). Rats were orally administered with saline, EPA or DHA for 8 weeks, with the dosage of EPA or DHA as described previously [49,50,51], and then the MWM and OFT were performed. One day after behavioral tests, animals were sacrificed, and hippocampal samples were collected on ice and quick-frozen in liquid nitrogen, then stored in a −80 °C refrigerator for further experiments.

### 4.2. Morris Water Maze

To evaluate spatial and learning memory, a tank (150 cm in diameter) was filled with water at 25 ± 1 °C. A 10 cm-diameter platform was submerged 2 cm underwater. On the first trial day, the start positions varied randomly in four quadrants without repetition. Four trials were arranged for each day to allow the rats to reach the platform. If the rats failed to find the platform within 1 min, they were gently guided to the platform to stay for 15 s. The latency to reach the platform was recorded for each animal. On the fourth day, the platform was relocated to a different quadrant in the maze and the latency to locate the platform was recorded. On the last day, the platform was removed, and the rats were allowed to swim freely for 1 min. The latency to the platform, the time and the number of entries into the target quadrant were recorded by video track. The data was processed by the SuperMaze behavior analysis system (Shanghai Xinruan Information Technology Co., Ltd., Shanghai, China).

### 4.3. Open Field Test

The OFT was popularly used for rodent locomotor and exploratory behavior in a novel environment [52]. Round open field apparatus (diameter 100 cm, high 50 cm) with a white painted wall and floor was used, in which the animal total distance was recorded by a digital camera within 3 min.

### 4.4. Brain n-3/n-6 PUFA Analysis by Gas Chromatography-Mass Spectrometry

Three n-3 PUFAs, including EPA, docosapentaenoic acid (DPA) and DHA, and two n-6 PUFAs including cis-8,11,14-eicosatrienoic acid and AA, were profiled by GC-MS (Agilent, Santa Clara, CA, USA). The lipid extraction method in this study was referred to by Folch [53]. Briefly, the brain tissues were homogenized in chloroform/methanol solution (2/1), vortexed with 0.9% NaCl solution, and centrifuged at 500 rpm for 20 min. The underlayer phase was collected and dried with a nitrogen blower. To make the fatty acid methyl derivatization, the precipitates were incubated with 1.5% sulfuric acid methanol and methylene chloride solution at 100 °C for 1 h. Hexane and saturated sodium chloride solution were added and centrifuged at 500 rpm for 2 min after cooling. Finally, the hexane phase was separated and then dried by a nitrogen blower for gas chromatography–mass spectrometry (GC-MS) analysis. The GC-MS analysis was performed on InterCap Pure-WAX Capillary columns (30 m × 0.25 mm, 0.25 µm) utilizing helium as a carrier gas at a flow rate of 1.0 mL/min. The mass spectrometry was recorded at a proton energy of 70 eV.

### 4.5. Cytokine Detections

The pro-inflammatory cytokines (IL-1β, IL-6, and TNF-α) and anti-inflammatory factors (IL-4 and IL-10) in the hippocampus were quantified by the enzyme-linked immunosorbent assay (ELISA) kits according to the manufacturer’s instructions. The hippocampal tissues were homogenized in 1 × PBS solution (pH 7.4) at a ratio of 1:9 and centrifuged at 2000 rpm for 15 min at 4 °C. The supernatant was collected for quantification.

### 4.6. Real-Time Quantitative Polymerase Chain Reaction Analysis

The total RNA extraction was performed according to the manufacturer’s instructions. Briefly, the hippocampus was homogenized with 1 mL TRIzol reagent (15596026, Invitrogen, Carlsbad, CA, USA) and incubated for 10 min at room temperature (RT). Chloroform (200 µL) was added and mixed by vigorous shaking for 30 s and standing for 10 min at RT. The RNA precipitation was obtained after centrifugation (12,000 rpm for 15 min) and washed once with 75% ethanol. The precipitates were finally dissolved in 20 µL diethylpyrocarbonate (DEPC) water. A measure of 1 µg of total RNA was used to perform reverse transcription by the QuantiTect Reverse Transcription Kit (Vazyme, Nanjing, China), and real-time PCR was performed with AceQ Universal SYBR qPCR Master Mix (Vazyme, China) on an ABI7500. The amplification reaction condition was set as follows: denaturation at 95 °C for 10 min, followed by 40 cycles of denaturation at 95 °C for 10 s and annealing/extension at 60 °C for 30 s. The expression of fold changes for target genes was calculated by the 2^−ΔΔct^ method. The primer sequences were listed in Table 1.

### 4.7. Western Blotting Analysis

The total hippocampus protein was extracted by RIPA lysis buffer containing 1 mM phenylmethanesulfonyl fluoride (PMSF) on ice and quantified using a BCA protein assay kit. Each sample (30 µg total protein) was separated by 12% sodium dodecyl sulfate-polyacrylamide gel electrophoresis (SDS-PAGE) and transferred onto polyvinylidene fluoride (PVDF) membranes. The membranes were blocked with Tris-buffered saline containing 5% skim milk for 2 h at RT and incubated with primary antibodies at 4 °C overnight. Subsequently, the membranes were washed three times with TBST (0.1% Tween-20) and incubated with HRP-labeled secondary antibodies for 2 h at RT. The bands were detected using an enhanced chemiluminescence (ECL) system and analyzed by Image J Software. The primary antibodies used were as follows: iNOS (1:500, sc-7271, Santa Cruz, CA, USA), CD206 (1:500, sc-70586, Santa Cruz, CA, USA), GFAP (1:500, sc-33673, Santa Cruz, CA, USA), C3 (1:500, sc-28294, Santa Cruz, CA, USA), proBDNF (1:500, sc-65514, Santa Cruz, CA, USA), TrKB (1:500, sc-377218, Santa Cruz, CA, USA), BDNF (1:1000, 28205-1-AP, proteintech, Wuhan, China), p75 (1:500, sc-271708, Santa Cruz, CA, USA) and β-actin (1:1000, #4967, CST, Danvers, MA, USA). The secondary antibodies were HRP-conjugated anti-mouse (1:1000, #7076, CST, MA, USA) and HRP-conjugated rabbit (1:1000, #7074, CST, MA, USA).

### 4.8. Statistical Analysis

The data were expressed as the mean ± standard error (SEM) and analyzed by GraphPad Prism (GraphPad Software 9.0.0, San Diego, CA, USA). Statistical comparisons were performed using one-way analysis of variance (ANOVA) followed by Fisher's LSD or Tukey's post hoc test. Statistical significance was considered as *p* < 0.05.

## Figures and Tables

**Table 1:** The primer sequences of target genes.

Genes	Forward Primer	Reverse Primer
BDNF	F:5′-CAAAAGGCCAACTGAAGC	R:5′-CGCCAGCCAATTCTCTTT
TrkB	F:5′-CACACACAGGGCTCCTTA	R:5′-AGTGGTGGTCTGAGGTTGG
p75	F:5′-TGCTCCATTTCCATCTCAG	R:5′-GATAGGTCCGTAATCCTCTTC
iNOS	F:5′-TGGAGCGAGTTGTGGATTGT	R:5′-GTAGTGATGTCCAGGAAGTAGGT
CD206	F:5′-GTTTCCATCGAGACTGCTGC	R:5′-GCCACTTTCCTTCAACATTTCG
ARG-1	F:5′-GGTAGAGAAAGGTCCCGCAG	R:5′-CAGACCGTGGGTTCTTCACA
C3	F:5′-TGTGGGTGGATGTGAAGGAC	R:5′-CTTGTCCACAGCCACTAGCC
IBA1	F:5′-CAACAAGCACTTCCTCGATGATC	R:5′-TGAAGGCCTCCAGTTTGGACT
GFAP	F:5′-CCAAGATGAAACCAACCT	R:5′-CGCTGTGAGGTCTGGCTT
S100B	F:5′-CTCTGTCTACCCTCCTAGTCC	R:5′-GACATCAATGAGGGCAACCAT
S100A10	F:5′-TATCACTAGTGGCGGGGCTC	R:5′-ATCAAGGTGTGGGTACCAGG
β-actin	F:5′-ACGGTCAGGTCATCACTATCG	R:5′-GTTTCATGGATGCCACAGGATT

## Data Availability

All data are contained within this article.
